# Ebb and Tide of Glucokinase

**DOI:** 10.1155/EDR.2001.168

**Published:** 2001

**Authors:** Franz M. Matschinsky

**Affiliations:** 1 Diabetes Center and Department of Biochemistry and Biophysics University of Pennsylvania School of Medicine Philadelphia PA 19104 USA


### Editorial: Ebb and Tide of Glucokinase

FRANZ M. MATSCHINSKY

Diabetes Center and Department of Biochemistry and Biophysics, University of Pennsylvania School of Medicine, Philadelphia, PA 19104, USA; matsch@mail.med.upenn.edu

This journal's editors have asked me to comment on the paper in this issue by Jetton et al. entitled "Substrate induced nuclear export and peripheral compartmentalization of hepatic glucokinase correlates with glycogen deposition." The investigations of these authors exemplify the remarkable recent trend in studies of intermediary metabolism paying careful attention to the precise intracellular location of biochemical processes including also those that are conventionally assumed to occur in the aequeous cytoplasmic compartment. The investigative history of the glucokinase enzyme—the topic of the paper editorialized here—strikingly illustrates the slow pace of evolution in studies of this particular aspect of intermediary metabolism, starting perhaps with the pioneering concepts of Paul Srere in the sixties [1].

Glucokinase was long considered a typical soluble cytosolic enzyme. Sidney Weinhouse and Alberto Sols discovered the enzyme in the high speed supernatant of liver extracts with negligible residue in the particulate fraction [2,3]. This distribution contrasted with that of glucose-6-phosphatase which was associated with the microsomes. A striking intraacinar gradient of glucokinase exhibiting significantly higher activities in the pericentral as compared to the peripheral zones was discovered in the late seventies [4]. It is of interest that glucose-6-phospahatase has an intraacinar gradient opposite to that of glucokinase. The kinetic characteristics of the enzyme, its S0.5 of about 8.0 mM, ATP Km of about 0.3 mM and Hill coefficient of about 1.7 explained its optimal operation in the aequeous cytosolic compartment of hepatocytes where physiological glucose levels of 5 to 8 mM approximate those of plasma and $ATP^{2-}$, the second substrate, is about 2.5 mM [5]. Consequently, a soluble glucokinase could operate close to its inflection point of about 4.0 mM where it is most responsive to changes of glucose levels. Since the enzyme is not directly controlled by glucose-6-phosphate as feedback inhibitor—more about this later—regulation of its activity was initially thought to be primarily due to alteration of protein synthesis stimulated by insulin. After glucokinase was discovered in pancreatic islets the conceptualization of the enzyme as cytoplasmic beta-cell glucose sensor followed the general outline of its biochemistry in hepatocytes except that its expression was considered to be controlled directly by glucose rather than by insulin [5].

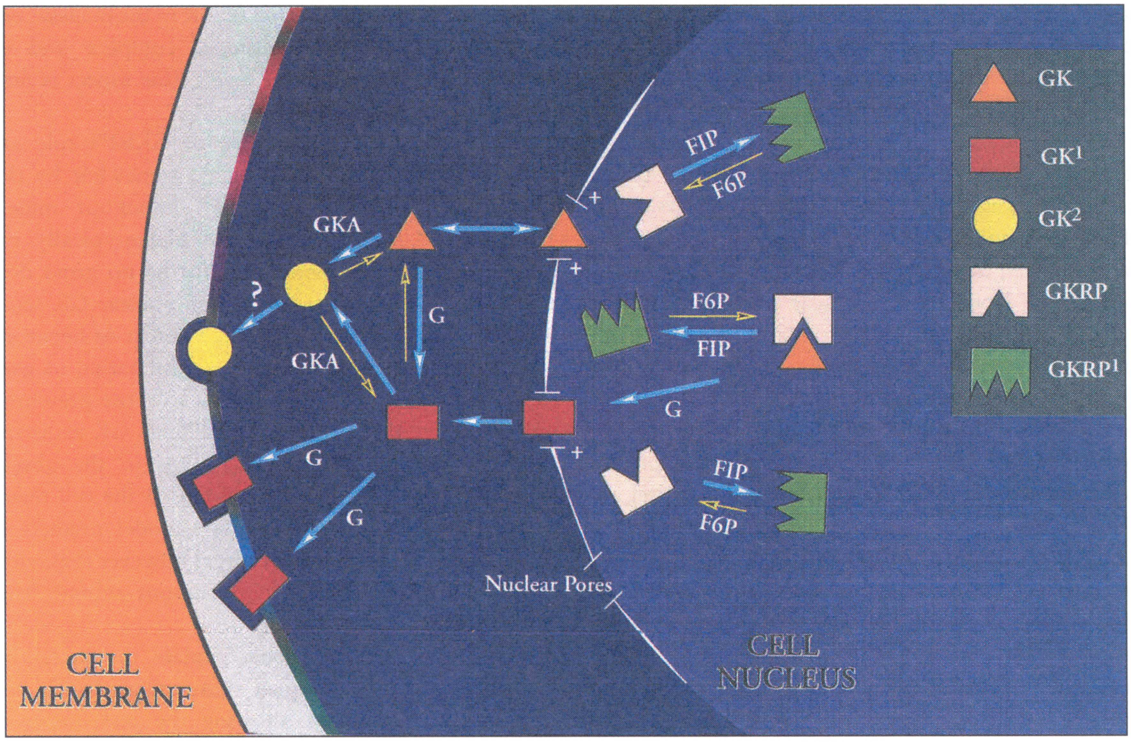

**FIGURE 1**

**MOLECULAR BASIS OF THE INTRACELLULAR EBB AND TIDE OF GLUCOKINASE**

The nuclear and cytosolic compartments, nuclear membrane pores and cell membrane associated GK binding sites are sketched. Three interconvertable GK forms are postulated to exist (i.e. GK, $GK^1$, and $GK^2$ depicted by distinct ikons). They are in equilibrium and GK predominates in the basal state. This equilibrium is shifted by glucose, pharmacological (and putative endogenous) GK activators and by GKRP. F6P and F1P influence the equilibrium between a GK inhibitory form (GKRP) and non inhibitory form ($GKRP^1$) of the nuclear protein. Nuclear and cytosolic GK and $GK^1$ are in equilibrium and are able to traverse the nuclear membrane through its pores. Structural elements associated with the cell membrane sequester $GK^1$ and $GK^2$ via specific binding sites which are exposed by glucose or GK activator binding.

The "classic picture," as briefly outlined above, has been replaced step by step by a dynamic "modern view" in which subcellular compartmentation and allosteric regulation add a higher level of complexity and the possibility of control on a time scale of seconds to minutes. This development is greeted with a sense of excitement in this sector of metabolic regulation research. The present report by Jetton et al. has to be read with such a mind set.

A few historical and prospective comments about these recent discoveries may aid in the understanding of this important topic. The seminal discovery of the glucokinase regulatory protein (GKRP) in the late 1980s by van Schaftingen got the ball rolling. GKRP is a 68.5 KD protein that inhibits glucokinase competitively with glucose [6]. Its efficacy is increased by fructose-6-phosphate (F6P) and diminished by fructose-1-phosphate (F1P) explaining the physiologically and potentially therapeutic relevant activation of glucose metabolism by fructose. It is noteworthy that GKRP regulation of glucokinase does not seem to be a factor in pancreatic beta-cell glucose metabolism. The work of Agius, Miwa, Mookhtiar and Toyoda opened an entirely new dimension by demonstrating the nuclear localization of GKRP and its role in glucokinase redistribution between nuclear and cytosolic compartments, the nutrient regulated intracellular "ebb and tide" of glucokinase [7,8]. The newest extension of this dynamic view is seen in the present and related publications demonstrating quite convincingly that glucokinase associates reversibly with the glycogen synthesis apparatus as it initiates the postprandial process of glucose polymerization at sites close to the cell membrane of the hepatocyte.

Recent observations suggesting an association of glucokinase with the insulin containing secretory granules are relevant in this context [9]. Evidence for glucose responsive circumscribed polar sequestration of beta cell glucokinase was first reported by Bonner-Weir and her group and the number of publications confirming and extending these earlier observations has steadily grown increasing the weight of the evidence. A physiological role, if any, for insulin granule associated glucokinase has, however, not been established or conceptualized.

The nutrient dependent subcellular compartmentation and dynamic ebb and tide of glucokinase in hepatocytes (and possibly pancreatic beta-cells) has profound implications for the biochemistry and molecular pathology of this enzyme and entices the writer of this editorial to sketch a minimal working model of this particular aspect of glucokinase regulation (Figure 1). Central to this model are the ligand induced slow transition (LIST) of glucokinase structure due to conformational changes of the enzyme as first demonstrated by Kenneth Neet [10] and the existence of multiple allosteric regulatory sites which interact with activators, inhibitors and cellular matrix proteins, many of them hypothetical at this juncture. Glucose is the prime mover in this model and fructose serves as physiologically significant indirect modifier via F1P, consistent with the data (see, for example, Figure 1 of Jetton's paper). An allosteric activator site for a putative endogenous stimulator is postulated by extrapolating from recent reports of activating mutations found in patients with persistent hyperinsulinemic hypoglycemia in infancy (PHHI) and from successful pharmacological activation of glucokinase [11,12]. Glucose generates the activated $GK^1$ form which dissociates from the GKRP/GK complex and is able to exit through nuclear pores possibly by involving specific recognition sequences of the enzyme. $GK^1$ has also increased affinity for matrix ankers close to the cell membrane (e.g. actin filaments or other ultramicroscopic structures). It is also speculated here that the binding might enhance the specific activity and increase the glucose affinity of the enzyme. $GK^1$, now positioned strategically, generates glucose-6-phosphate (G6P) locally enhancing the efficacy of the cell membrane associated glycogen apparatus. Fructose via F1P dissociates the GKRP/GK complex by generating $GKRP^1$ and GK is able to exit through nuclear pores. By mass action $GK^1$ in the cytosol increases and hence the binding of $GK^1$ to the glycogen synthesis apparatus is enhanced even at relatively low glucose. Lowering the cellular fructose or glucose reverses the process. The new class of allosteric activators (GKAs) facilitates the production of G6P by inducing the activated $GK^2$ form which may or may not be matrix bound. It is conceivable that these activators could counteract F6P dependent GKRP inhibition of GK in the nucleus, enhance dissociation of the GKRP/GK complex and elevate nuclear and cytosolic $GK^2$ levels (this aspect is not illustrated in the diagram).

The biological significance of GKRP's role and the sketch drawn here are mitigated somewhat by the outcome of studies with GKRP knockout mice [13]. Surprisingly, these animals are phenotypically normal. Most importantly, there seems to be no gross defect of glucose homeostasis, even though nuclear GK sequestration is abrogated and total GK content of hepatocytes is reduced by ca 50%. It will be illuminating to use these knockout mice in studies similar to those presented here by Jetton et al. to study intracellular GK redistribution during fructose and glucose loads in the absence of GKRP. Does the movement of GK to the cell membrane depend on nuclear GKRP? The model predicts that it would not. One should not forget however that, viewed in isolation, studies with gene knockout animals might be misleading.

This simple model could explain the currently published liver data. It implies that activated $GK^1$ has new binding sites exposed allowing the attachment of the enzyme to cellular matrix components. It also implies the possibility of point mutations of GK that might specifically interfere with this process and might be a cause of MODY-2 or even permanent neonatal diabetes mellitus (PNDM) [14]. It is noteworthy in this context that the diabetogenic effect of certain GK mutations in MODY-2 remains unexplained. The model raises the possibility of impaired or enhanced intracellular GK redistribution which might be caused by pathological changes of the components that participate in this process but would not be GK linked, i.e. are possible candidates for MODY-X. The full characterization of GK missense mutations causing PHHI, MODY-2 and PNDM will therefore require the assessment of sequences that must be postulated to govern enzyme compartmentation. Incidently, much of what has been discussed here may also pertain to the pancreatic beta-cell, with modifications, of course, because GKRP is probably absent.

Certain spontaneous and man made mutants of GK may represent the fully activated state depicted here as $GK^2$ [11]. They have a kcat of as much as 1.5 times normal, a glucose So.5 of 1.0 to 2.5 mM instead of 8.5 mM and lower Hill coefficients (a measure of the cooperativity with glucose). It is conceivable that mutant induced changes of the enzyme influence its subcellular distribution that occurs physiologically. It seems plausible to predict that certain activated GK forms would enhance translocation to the cell membrane. Individuals with such mutations (i.e. V455M) maintain significant glycogen stores in the face of low bloodsugar of about 2.5 mM as shown by the results of glucagon challenges. GK activated by allosteric mutants offers a unique opportunity for studying the biochemistry of intracellular movements of enzyme.

These are just a few of the implications of the modernized view of GK biochemistry as exemplified by the paper of Jetton et al. It can be expected that the continued pursuit of research focusing on the dynamics of intracellular hepatic (or beta-cell) GK distribution as influenced by nutritional, pharmacological and genetic factors will greatly expand our understanding of glucose homeostasis in health and will assist in the therapeutic management of diabetes mellitus and related disorders.

### References

1. Eastabrook R., Srere P., Eds. (1978). Microenvironments and Metabolic Compartmentalization. Academic Press, New York.
2. Weinhouse, S. (1976). Regulation of glucokinase in the liver, *Curr. Top. Cell. Regulation*, 11, 1.
3. Salas J., Salas M., Vinuela E., Sols A. (1965). Glucokinase of rat liver: purification and properties, *J. of Biol. and Chem.*, 240, 1014.
4. Trus M., Zawalich K., Gaynor D., Matschinsky F. M. (1980). Hexokinase and glucokinase distribution in the liver lobule, J. of Histochem. and Cytochem., 28, 579.
5. Matschinsky F. M. (1996). Banting Lecture 1995: A lesson in metabolic regulation inspired by the glucokinase glucose sensor paradigm, *Diabetes*, 45, 223.
6. Van Schaftingen E., Dettheu M., Veiga-da-Cunha M. (1994). Short-term control of glucokinase activity: Role of a regulatory protein, *FASEB Journal*, 8, 414
7. Van Schaftingen E., Veiga-da-Cunha M., Gerin I., Moukil M. (2000). Control of glucose phosphorylation/dephosphorylation in liver, in Molecular Pathogenesis of MODYS, F. M. Matschinsky, M. A. Magnusen eds., Karger Verlag, 136.
8. Agius L., Peak M. (1993). Intracellular binding of glucokinase in hepatocytes and translocation by glucose, fructose and insulin, *Biochem. J.*, 296, 785.
9. Stubbs M., Aiston S., Agius L. (2000). Subcellular localization, mobility and kinetic activity of glucokinase in glucose-responsive insulin-secreting cells, *Diabetes*, 49, 2048.
10. Neet K. E. (1996). Cooperativity in enzyme function: Equilibruim and kinetic aspects, in Contemporary Enzyme Kinetics and Mechanisms, D. L. Prurich ed., Academic Press, 133.
11. Glaser B., Kesavan P., Heyman M., Davis E., et al. (1998). Familial hyperinsulinism caused by an activating glucokinase mutation, *N. Eng. J. of Med.*, 338, 226.
12. Grimsby J., Sarabu R., Bizzarro F. T., Coffey J. W., et al. (2001), Abstract #460-P, Diabetes 50 (Supplement 2).
13. Grimsby J., Coffey J. W., Doorozniak M. T., Magram J., et. al. (2000). Characterization of glucokinase regulatory protein deficient mice, *J. of Biol. Chem.*, 275, 7826.
14. Njolstadt P. R., Sovik O., Cuesta-Munoz A., Bjorkhauk L., et. al. (2001). Neonatal diabetes mellitus due to complete glucokinase deficiency, *N. Eng. J. of Med.*, 344, 1588.

